# Tourette syndrome: current data, comorbidities, and therapeutic approach in children

**DOI:** 10.1186/1824-7288-40-S1-A68

**Published:** 2014-08-11

**Authors:** R Rizzo

**Affiliations:** 1 Child and Adolescent Neurology and Psychiatry, Department of Medical and Pediatric Science; 2 Catania University, Via S.Sofia 78 Catania, 95123 Italy

## 

Tourette syndrome (TS) is the primary tic disorder with an estimated prevalence close to 1% between 5 and 18 years of age [1]. Motor and phonic tics are the core features of TS [2]. In addition to their well-characterized phenomenology, tics display a peculiar variability over time, which is strongly influenced by a variety of contextual factors. A relevant proportion of patients with TS display complex, tic-like, repetitive behaviors that include echophenomena, coprophenomena, and nonobscene socially inappropriate behaviors (NOSIBs). Co-morbid conditions are attention deficit hyperactivity disorder (ADHD), obsessive compulsive behaviours/disorder (OCB/D) and autistic spectrum disorder (ASD); co-existent psychopathologies include depression, anxiety, oppositional defiant disorder (ODD), conduct disorder (CD) and personality disorders (PDs) [3]. The complexity of the Tourette spectrum has been confirmed by cluster and factor analytical approaches [4]. It is suggested that TS is not a unitary condition and that one phenotype ("Pure TS" [tics only]) occurs in about 10-14 % [5]. The presence of comorbid attention deficit hyperactivity disorder (ADHD) is the main determinant of cognitive dysfunction in TS patients and influences heavily also the risk of developing disruptive behaviors [6].The burden of behavioral comorbidities is very important in determining significant impairment, poor self-esteem, and a low quality of life [7,8].While the evidence for a genetic contribution is strong, several genes, including *SLITRK1*, LIM homeobox (*LHX6*, *LHX8*), and *HDC* have been suggested to be responsible for the different clinical phenotypes [9,10].However its exact nature has yet to be clarified fully. Aetiological factors include genetic vulnerability pre- and peri-natal difficulties (PNDs), and probably neuro-immunological factors. Neuro-imaging are helpful to exclude other conditions, and although abnormalities are described, in an individual patient, they are not diagnostic. Treatment includes psycho-education and reassurance, medications, target-specific botulinum toxin injections [11] and in a few severe refractory adult cases, deep brain stimulation life [12].This review will summarise and highlight selected main findings from the author's clinic.
